# Pelvic floor dysfunction in postpartum women: A cross-sectional study

**DOI:** 10.1371/journal.pone.0308563

**Published:** 2024-10-03

**Authors:** Qian Gao, Mingbo Wang, Jie Zhang, Yangzhe Qing, Ziyi Yang, Xin Wang, Xujuan Xu, Qing Ye, Feng Zhang

**Affiliations:** 1 Department of Nursing, Affiliated Hospital of Nantong University, Nantong, Jiangsu Province, China; 2 School of Nursing and Rehabilitation, Nantong University, Nantong, Jiangsu Province, China; 3 Medical school of Nantong University, Nantong, Jiangsu Province, China; Iran University of Medical Sciences, ISLAMIC REPUBLIC OF IRAN

## Abstract

**Background:**

Pelvic floor dysfunction (PFD) is a disease of weakened pelvic floor support tissues, leading to changes in the pelvic organ position and function of pelvic organs, with long-term effects on women. This study aimed to assess pelvic floor function using electrophysiology and clinical symptoms, exploring the risk factors for PFD one month postpartum.

**Methods:**

This cross-sectional study included 845 women from postpartum outpatient clinic of Nantong Affiliated Hospital from August 2019 to October 2021. Pelvic floor muscle strength was evaluated via pelvic floor surface electromyography. Clinical symptoms (urinary incontinence (UI) and pelvic organ prolapse) were diagnosed by gynecologists. Sociodemographic, pregnancy, and obstetrical data were obtained from self-reported questionnaires and electronic records.

**Results:**

The study identified maternal age, parity, immigrant status, and economic income as factors were related to PFD. Gestational constipation increased the risk of abnormal resting muscle strength (OR:1.553, 95%CI: 1.022–2.359). Cesarean delivery was associated with higher rates of abnormal resting muscle strength than vaginal delivery (post-resting stage: OR, 2.712; 95% CI, 1.189–6.185), but a decreased incidence of UI (OR: 0.302; 95% CI, 0.117–0.782). Increased gestational weight gain was correlated with a greater risk of developing UI (OR:1.030, 95%CI: 1.002–1.058). Women with vaginal inflammation faced a higher risk of abnormal fast-twitch muscle (OR: 2.311, 95%CI: 1.125–4.748).

**Conclusions:**

In addition to uncontrollable factors like mode of delivery, age, and parity, interventions targeting weight gain and constipation during pregnancy and vaginal flora could mitigate the risks of PFD. Educational programs for pregnant women should emphasize a proper diet and lifestyle. For women with vaginal inflammation, clinical treatment should be carried out as soon as possible to avoid further aggravating the damage to the pelvic floor muscles.

## Introduction

The female pelvic floor is a complex pelvic floor support system consisting of various layers of muscles, fascia, ligaments, and nerves that interact to maintain the normal position of pelvic organs and support sexual function [1]. Pelvic floor dysfunction (PFD) occurs when these support tissues weaken, leading to changes in the pelvic organ and function [2]. PFD encompasses conditions such as urinary incontinence (UI), pelvic organ prolapse (POP), and sexual dysfunction [3].

PFD has long-term effects on women. Untreated UI can profoundly impact the lives of women due to its uncontrollable nature. Women with PFD often experience psychological challenges, including fatigue and sleep disorders over time [4]. Furthermore, the difficulties in daily life associated with PFD can hinder postpartum women from fully embracing their maternal role and may contribute to breastfeeding difficulties [5]. In addition, PFD is associated with decreased sexual function, including decreased arousal, fewer frequent orgasms, and dyspareunia [6]. Postpartum women who experience urinary leakage are more likely to show reluctance or reduce the frequency of intercourse [7]. Therefore, it is crucial to emphasize the importance of pelvic floor muscle (PFM) function in women.

Various factors have been identified that increase the susceptibility to PFD. Epidemiological research demonstrates that age, constipation, obesity, and menopause influence PFM strength [8]. Additionally, lifestyle factors such as smoking, alcohol consumption, coffee and tea intake, and level of physical activity also contribute to PFD [9]. Pregnancy and childbirth are well-established independent risk factors for PFD [10],with the prevalence of pelvic floor trauma notably elevated increasing immediately postpartum due to the strain on the pelvic floor during pregnancy and childbirth. Birth-related factors such as parity, mode of delivery, use of oxytocin, fetal weight, and prolonged second stage of labor significantly elevate the risk of PFD [11]. The effect of the mode of delivery on pelvic floor function remains controversial and there is limited research exploring the influence of maternal lifestyle and vaginal flora on PFD.

Currently, while numerous articles investigate the risks of affecting maternal PFM function, research on these factors remains incomplete. There is a continued need for studies on PFD. This study aims to comprehensively explore the risk factors influencing PFMs in greater depth. Specifically, we utilize electrophysiological measurements and clinical diagnosis to investigate these factors in PFD.

## Materials and methods

This was a cross-sectional observational study. The convenient sampling method was conducted at the Affiliated Hospital of Nantong University in China between August 2019 and October 2021. This study was approved by the Ethics Committee of Affiliated Hospital of Nantong University (2019-K078-01).

### Participants

This study recruited women who underwent routine check-ups at the maternal and Child Health Care Center six to eight weeks postpartum. During recruitment, the investigator provided detailed information on the research purpose and content to the respondents. Data collection only proceed after both parties confirmed and signed the informed consent form. The inclusion criteria were as follows:1) older than 18 years old; 2) singleton pregnancy; 3) six to eight weeks postpartum; 4) cessation of lochia; and 5) achieved current and previous obstetrical delivery information through medical records. The exclusion criteria encompassed: 1) malignancies; 2) epilepsy; 3) severe vaginal or intestinal infections; 4) clinical diagnosis of PFD before or during pregnancy, and 5) use of cardiac pacemakers.

### Pelvic floor function assessment

#### The measurement of pelvic surface electromyography (EMG)

**Fig 1:**
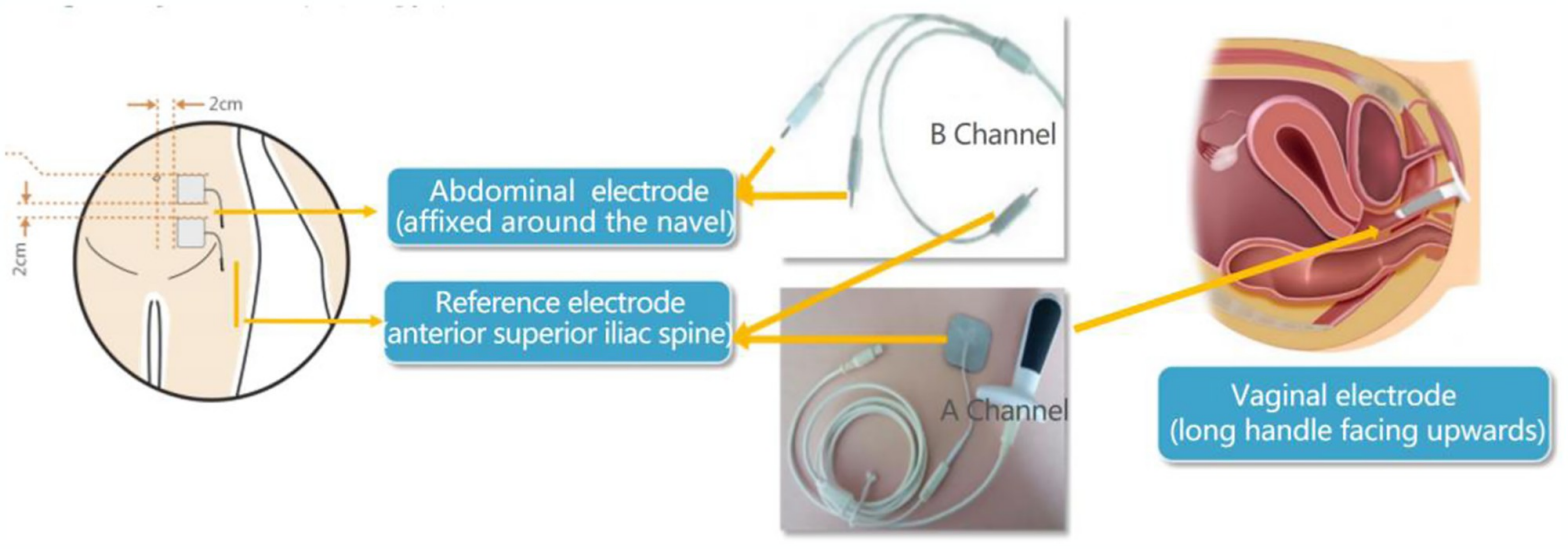
Schematic diagram of electrophysiological examination operation.

Surface EMG of the PFMs was utilized to access the neural activity, serving as an indicator of early detection of PFD [12]. In this study, the pelvic floor surface EMG analysis was conducted using MLD A2 system (Nanjing Medlander) to evaluate the pelvic floor function. The analysis included four stages (pre-rest, fast-twitch muscle contraction, slow-twitch muscle contraction, and post-rest stages), specifically tailored for the women six to eight weeks postpartum. The pre-rest stage measured the baseline of the PFMs, with a normal value of 2 to 4μV and the variability of less than 20%. During the fast-twitch muscle contraction stage (type II fibers), normal peak values ranged from 35 to 45μV, assessing dynamic muscle strength and reaction speed. The slow-twitch muscle contraction (type I fibers) stage tested the stability of dynamic slow-twitch muscle strength and contractile control. Normal contraction amplitude ranged from 25 to 30μV, with a variability of less than 20%. The post-rest stage measured static PFM tone after activity, with a normal value of 2 to 4μV. After emptying the bladder, participants were instructed to lie supine on the stretcher with their lower limbs flexed. During the test, two electrode probes were placed on the abdomen and vaginal opening, respectively. The obstetrician instructed the participants how to contract and relax pelvic muscles to control the rise and fall of a curve displayed on the system. The process included four stages: the pre-rest (no movement), the fast-twitch muscle contraction (five one-second contractions), the slow-twitch muscle contraction (five 10-second sustained contractions with five-second rests), and the post-rest (rest stage after exercises). Each stage was deemed abnormal if measurements fell outside normal ranges. The entire procedure was overseen by medical professionals, as shown in Table 1, and the specific placement is shown in Fig 1.

**Table 1:** Normal range of values for each phase of electrophysiological testing.

Stage name	Parameter name	Reference range
Pre-rest stage	Mean	<4μV
	Variability	<0.2
Post-rest stage	Mean	<4μV
	Variability	<0.2
Fast muscle stage	Maximum value	>40μV
	Rise Time	<0.5s
	Recovery Time	<0.5s
Slow muscle stage	Mean	>35μV
	Variability	<0.2

#### Clinical diagnosis of PFD (POP and UI)

POP is defined as the abnormal position and dysfunction of pelvic organs caused by abnormal pelvic floor muscles and fascia tissues. The primary symptom of POP is the protrusion of the vaginal mass, often accompanied by issues such as urination, defecation, and sexual dysfunction. Clinically, POP is diagnosed when the lowest point of prolapse reaches or exceeds the hymen or based on a POP quantitation index of ≥ II degree [13].

UI encompasses any involuntary urine loss and is categorized into stress UI, urgency UI, and mixed UI [14]. Stress UI is diagnosed as symptoms of urine leakage caused by increased abdominal pressure which was caused by activities such as sneezing, coughing, exercise, or changes in posture. Urgency UI is diagnosed on the basis of symptoms of urinary leakage that occur along with a sense of urgency and a sudden urge to urinate that cannot be postponed. Mixed UI is a combination of stress incontinence and urgency urinary incontinence. In our study, due to the small proportion of UI in the parturient, we did not divide the analysis according to the category of UI.

The evaluation and diagnosis of UI and POP, and the pelvic floor surface electromyography test were conducted by obstetricians.

#### Potential factors of PFD

The survey contents covered various demographic and health factors, including age, occupation, economic income, education level, whether immigrant population, marriage, intake of tea, coffee, family history of pelvic floor muscle disorders, history of endoscopic surgery, pre-pregnancy body mass index, gestational diabetes, gestational weight gain, pregnancy complications, constipation during pregnancy, mode of delivery, perineal laceration, lateral episiotomy, vaginal pus cells, vaginal bacteria, gestational weeks, parity, fetal macrosomia. Maternal demographic information, delivery outcomes, pregnancy complications, and neonatal outcomes were collected from medical records. A self-report questionnaire was administered to mothers during their outpatient visit 42 days postpartum by a nurse. Follow-up medical records were retrieved to collect delivery data and verify maternal self-reported medical history. The detailed investigation information can be found in S1 Table.

Constipation was evaluated by the validated Rome IV criteria, which consists of six questions about the presence or absence of 1) straining during more than 1/4 of defecation; 2) irregular or hard stools more than 1/4 of defecation; 3) sensation of incomplete evacuation more than 1/4 of defecation; 4) sensation of anorectal blockage more than 1/4 of defecation; 5) manual maneuvers to facilitate more than 1/4 of defecation; or 6) fewer than three spontaneous defecations per week. A diagnosis of constipation was made if a woman experienced at least two of these symptoms for a minimum of three months [15].

Pus cells and miscellaneous bacteria were diagnosed by vaginal leucorrhea test. During the outpatient examination six to eight weeks after delivery, the gynecologist performed a leucorrhea test. After the women had emptied her urine and set up the bladder lithotomy position, the gynecologist used a sterile cotton swab to collect vaginal secretions in the upper vagina or areas with obvious lesions. During the collection process, gynecologist avoided touching the vaginal wall and cervix to prevent bleeding from affecting sample quality. Vaginal secretions were examined using both wet film and stained smears under an electron microscope. The cleanliness of the vagina was categorized into four grades: 1) “zero,” indicating numerous epithelial cells with few or no bacteria and zero to five white blood cells per high-power field (HP); 2) “+,” indicating a moderate amount of epithelial cells and 5–15 white blood cells per HP; 3) “++,” indicating few epithelial cells and 15–30 white blood cells per HP; 4) “+++,” indicating few or no epithelial cells and more than 30 white blood cells per HP, which is more commonly associated with severe vaginitis.

### Statistical analysis

We calculated the sample size based on the primary outcome measure (abnormality incidence of electrophysiological indicators). In a previous study with similar population, the abnormal incidence of electrophysiological indicators was 47.6% [16]. As a result of accepting α error as 5% and δ allowance error as 4%, the sample size was calculated to be 599. Ultimately, this study required at least 600 samples.

In this study, data was entered through Epidata, and analyzed by SPSS 27.0. Two people entered the data separately, and the consistency analysis was performed in parallel. Normally distributed data were expressed as mean ± standard deviation, and differences between groups were expressed by parametric tests. Non-normally distributed data were expressed by frequency, and the difference between groups was selected according to the theoretical frequency of the chi-square test, corrected chi-square test, and Fisher’s exact probability analysis. Finally, binary logistic regression was used to explore the relationship between risk factors and postpartum pelvic floor electrophysiology, UI, and POP. In model A, we adjusted for demographic data, including age, occupation, economic income, education level, immigrant population. In model B. we controlled pregnancy-related information on the basis of model A, including pre-pregnancy body mass index, pregnancy diabetes, weight gain during pregnancy, pregnancy complications, and constipation during pregnancy. In model C, we adjusted for postpartum-related information on the basis of model B, including delivery mode, perineal laceration, lateral episiotomy, vaginal pus cells, vaginal bacteria, gestational weeks, parity, and fetal macrosomia.

## Results

This study surveyed 1022 women who attended a postpartum center. After excluding 26 cases of twin pregnancies, 13 cases of UI during pregnancy, two cases of prenatal POPs, eight cases of UI surgery, and 128 cases of prenatal leakages, a total of 845 women were included.

The average age of women in this study was 29.29 years (standard deviation [SD]: ±3.75). The majority of participants were married (n = 842, 99.6%), non-migrants (n = 762, 90.2%), and employed (n = 699, 82.7%). Furthermore, more than half of the women had obtained a bachelor’s degree or higher (n = 664, 78.6%). A minority of participants reported undergoing hysteroscopic surgery (n = 39, 4.6%), and a small proportion consumed took coffee (n = 68, 8%) or tea (n = 18, 2.1%) during pregnancy. (Table 2).

**Table 2:** General characteristic of postpartum women.

Characteristic	Number of cases(n = 845)	Composition (%)
Maternal age, (years)	29.29±3.75	-
Marital status		
Unmarried	2	0.2
Married	842	99.6
Divorced	1	0.1
Migrant population		
Yes	81	9.6
No	762	90.2
Maternal education, (years)		
≤9	50	5.9
10–12	80	9.5
13–16	664	78.6
≥17	51	6
Employment status		
Employed	699	82.7
Unemployed	146	17.3
Monthly income, (RMB/month)		
≤2000	30	3.6
2001–5000	411	48.6
≥5001	404	47.8
Family history of pelvic floor muscle disorder		
Yes	11	1.3
No	834	98.7
Consumption of tea		
Yes	18	2.1
No	827	97.9
Coffee intake		
Yes	68	8.0
No	777	92.0
Laparoscopic/hysteroscopic surgery		
Yes	39	4.6
No	806	95.4

### Risk factors for PFM electrophysiological indicators

Upon EMG diagnosis, a significant majority of women exhibited PFD, ranging from78 to91.5%. During the pre-rest stage, PFM function was associated with constipation during pregnancy, and mode of delivery (P<0.05). Fast-twitch muscle contraction also showed association with constipation during pregnancy and mode of delivery, while slow-twitch muscle contraction was similarly related to these factors. Furthermore, differences in mode of delivery were observed between groups during the post-rest stage, with further shown in Table 3.

**Table 3:** Data during pregnancy and postpartum based on pelvic floor muscle surface electromyography.

	*Pre-rest stage*			*Post-rest stage*			*Fast muscle stage*			*Slow muscle stage*		
Characteristic	Normal pre-resting stage	Abnormal pre-resting stage	*P*	Normal post-rest stage	Abnormal post-rest stage	*P*	Normal fast muscle stage	Abnormal fast muscle stage	*P*	Normal slow muscle stage	Abnormal slow muscle stage (n = 773)	*P*
	(n = 154)	(n = 691)		(n = 186)	(n = 659)		(n = 186)	(n = 659)		(n = 72)		
Prepregnant BMI,(kg/m^2^)			0.831			0.979			0.979			0.059
<30	150(97.40)	668(96.67)		180(96.7)	638(96.81)		180(96.77)	638(96.81)		67(93.06)	751(97.15)	
≥30	4(2.60)	23(3.33)		6(3.23)	21(3.19)		6(3.23)	21(3.19)		5(6.94)	22(2.85)	
Weight gain in pregnancy, (kg)	14.53±4.71	14.39±6.67	0.817	14.72±5.20	14.42±6.35	0.464	14.38±5.07	14.43±6.67	0.918	15.35±4.58	14.33±6.49	0.193
Pregnancy complications			0.637			0.132			0.263			0.509
Yes	55(35.71)	233(33.72)		72(38.71)	216(32.78)		57(30.65)	231(35.05)		22(30.56)	266(34.41)	
No	99(64.29)	458(66.28)		114(61.29)	443(67.22)		129(69.35)	428(64.95)		50(69.44)	507(65.59)	
GDM			0.737			0.185			0.257			0.328
Yes	17(11.04)	70(10.13)		24(12.90)	63(9.56)		15(8.06)	72(10.93)		5(6.94)	82(10.61)	
No	137(88.96)	621(89.87)		162(87.1)	596(90.44)		171(91.94)	587(89.07)		67(93.06)	691(89.39)	
Constipation during pregnancy			0.018			0.058			0.018			0.004
Yes	37(24.03)	234(33.86)		49(26.34)	222(33.69)		73(39.25)	198(30.05)		34(47.22)	237(30.66)	
No	117(75.97)	457(66.14)		137(73.66)	437(66.31)		113(60.75)	461(69.95)		38(52.78)	536(69.34)	
Gestational age, (week)			0.526			0.25			0.706			-
<37	9(5.84)	32(4.63)		12(6.45)	29(4.40)		10(5.38)	31(4.70)		3(4.17)	38(4.92)	
≥37	145(94.16)	659(95.37)		174(93.5)	630(95.60)		176(94.62)	628(95.30)		69(95.83)	735(95.08)	
Parity			0.199			0.547			0.754			0.955
1	103(66.88)	498(72.07)		129(69.35)	472(71.62)		134(72.04)	467(70.86)		51(70.83)	550(71.15)	
≥2	51(33.12)	193(27.93)		57(30.65)	187(28.38)		52(27.96)	192(29.14)		21(29.17)	223(28.85)	
Mode of delivery			<0.001			<0.001			<0.001			<0.001
Vaginal delivery	110(71.43)	371(53.69)		130(69.89)	351(53.26)		83(44.62)	398(60.39)		21(29.17)	460(59.51)	
Cesarean section	44(28.57)	320(46.31)		56(30.11)	308(46.74)		103(55.38)	261(39.61)		51(70.83)	313(40.49)	
Perineal laceration			0.586			0.516			0.242			0.616
Yes	76(69.09)	246(66.31)		90 (69.23)	232(66.10)		51(61.45)	271(68.09)		13(61.90)	309(67.17)	
No	34(30.91)	125(33.69)		40(30.77)	119(33.90)		32(38.55)	127(31.91)		8(38.10)	151(32.83)	
Lateral episiotomy			0.929			0.289			0.320			0.187
Yes	28(25.45)	96(25.88)		29(22.31)	95(27.07)		25(30.12)	99(24.87)		8(38.10)	116(25.22)	
No	82(74.55)	275(74.12)		101(77.69)	256(72.93)		58(69.88)	299(75.13)		13(61.90)	344(74.78)	
Fetal macrosomia			0.087			-			0.304			0.504
Yes	5(3.25)	9(1.30)		3(1.61)	11(1.67)		1(0.54)	13(1.97)		0(0)	14(1.81)	
No	149(96.75)	682(98.70)		183(98.39)	648(98.33)		185(99.46)	646(98.03)		72(100)	759(98.19)	
Vaginal pus cells			0.739			0.359			0.781			0.131
0-+	118(76.62)	538(77.86)		149(80.11)	507(76.93)		143(76.88)	513(77.85)		61(84.72)	595(76.97)	
++-++++	36(23.38)	153(22.14)		37(19.89)	152(23.07)		43(23.12)	146(22.15)		11(15.28)	178(23.03)	
Vaginal bacteria			0.493			0.985			0.057			0.588
0-+	136(88.31)	623(90.16)		167	592(89.83)		174(93.55)	585(88.77)		66(91.67)	693(89.65)	
				-89.78								
++-++++	18(11.69)	68(9.84)		19(10.22)	67(10.17)		12(6.45)	74(11.23)		6(8.33)	80(10.35)	

### Risk factors for clinical symptoms of PFD

When diagnosing PFD based on clinical symptoms rather than EMG, UI and POP were significantly less frequently identified. In our study, the incidence of UI significantly varied across groups with different modes of delivery (p<0.001), varying degree of vaginal pus cells (p = 0.001), and different degree of vaginal bacteria (p<0.001). Regarding POP, associations were found with mode of delivery (p = 0.003) and fetal macrosomia (p = 0.029). (Table 4).

**Table 4:** Data during pregnancy and postpartum, based on clinical symptoms.

	*Urinary incontinence*			*Pelvic organ prolapse*		
Characteristic	With urinary incontinence(n = 145)	Without urinary incontinence(n = 700)	*P*	With pelvic organ prolapse(n = 30)	Without pelvic organ prolapse(n = 815)	*P*
Prepregnant BMI, (kg/m^2^)			0.268			0.628
<30	143(98.62)	675(96.43)		30(100)	788(96.69)	
≥30	2(1.38)	25(3.57)		0(0)	27(3.31)	
Weight gain in pregnancy, (kg)	14.27±6.50	15.17±5.57	0.119	14.48±6.40	12.83±4.72	0.163
Pregnancy complications			0.619			0.93
Yes	52(35.86)	236(33.71)		10(33.33)	278(34.11)	
No	93(64.14)	464(66.29)		20(66.67)	537(65.89)	
GDM			0.222			0.801
Yes	19(13.10)	68(9.71)		4(13.33)	83(10.18)	
No	126(86.90)	632(90.29)		26(86.67)	732(89.82)	
Constipation during pregnancy			0.282			0.583
Yes	41(28.28)	230(32.86)		11(36.67)	260(31.90)	
No	104(71.72)	470(67.14)		19(63.33)	555(68.10)	
Gestational age, (week)			0.208			0.638
<37	10(6.90)	31(4.43)		2(6.67)	39(4.79)	
≥37	135(93.10)	669(95.57)		28(93.33)	776(95.21)	
Parity			0.979			0.338
1	103(71.03)	498(71.14)		19(63.33)	582(71.41)	
≥2	42(28.97)	202(28.86)		11(36.67)	233(28.59)	
Mode of delivery			<0.001			0.003
Vaginal delivery	112(77.24)	369(52.71)		25(83.33)	456(55.95)	
Cesarean section	33(22.76)	331(47.29)		5(16.67)	359(44.05)	
Perineal laceration			0.643			0.908
Yes	77(68.75)	245(66.40)		17(68.00)	305(66.89)	
No	35(31.25)	124(33.60)		8(32.00)	151(33.11)	
Lateral episiotomy			0.644			0.794
Yes	27(24.11)	97(26.29)		7(28.00)	117(25.66)	
No	85(75.89)	272(73.71)		18(72.00)	339(74.34)	
Fetal macrosomia			0.774			0.029
Yes	2(1.38)	12(1.71)		2(6.67)	12(1.47)	
No	143(98.62)	688(98.29)		28(93.33)	803(98.53)	
Vaginal pyocyte			0.001			0.307
0-+	98(67.59)	558(79.71)		21(70.00)	635(77.91)	
++-++++	47(32.41)	142(20.29)		9(30.00)	180(22.09)	
Vaginal bacteria			<0.001			0.974
0-+	116(80.00)	643(91.86)		27(90.00)	732(89.82)	
++-++++	29(20.00)	57(8.14)		3(10.00)	83(10.18)	

### Binary logistic regression on risk factors for postpartum pelvic floor dysfunction

After performing univariate analysis, we employed binary logistic regression to investigate the relationships between PFD and general information, pregnancy-related data, and postpartum factors separately. In model A, we observed that higher maternal age was associated with increased risk of UI (OR: 1.065, 95%CI:1.015–1.116) and abnormal fast muscle electrophysiology (OR: 1.048, 95%CI:1.001–1.)

In Model B, our findings indicated associations between migrant status and reduced muscle strength in slow-twitch muscle contractions (OR: 0.201; 95% CI: 0.046–0.872), as well as between age and UI (OR: 1.070; 95% CI: 1.019–1.123).:: Besides, a higher pre-pregnancy BMI was associated with a decreased risk of electrophysiological abnormalities in the slow-muscle contraction stage (OR:: 0.258, 95%CI: 0.088–0.759). Furthermore, our study revealed that constipation during pregnancy served as a risk factor for abnormal muscle strength during both pre- and post-resting stages (OR: 1.622, 95%CI: 1.079–2.437; and OR:1.470, 95%CI:1.014–2.437, respectively). Interestingly, constipation was found to be protective against abnormal fast-twitch muscle contraction (OR: 0.644; 95% CI: 0.456–0.908) and slow-twitch muscle contraction (OR: 0.435; 95% CI: 0.264–0.719).

The relationship between migrant population and slow muscle strength (OR: 0.211, 95%CI: 0.047–0.942),constipation during pregnancy and muscle strength at pre-resting stage (OR: 1.553, 95%CI: 1.022–2.359), age and UI (OR: 1.116, 95%CI:1.052–1.183), and constipation during pregnancy and fast/slow muscle strength (slow muscle stage: OR: 0.479, 95%CI: 0.285–0.804; fast muscle stage: OR: 0.652, 95%CI: 0.457–0930) observed in model B remained significant in model C. However, the association between pre-pregnancy BMI and PFD were not statistically significant in model C. Additionally, we found that maternal age was associated with fast-twitch muscle strength and muscle strength during the post-resting stage (fast muscle: OR: 1.073, 95%CI: 1.016–1.134; post-resting stage: OR: 0.937, 95%CI: 0.891–0.986). In model C, cesarean delivery increased the risk of abnormal surface muscle strength in the post-resting stage (OR:2.712, 95%CI: 1.189–6.185), but decreased the incidence of UI (OR: 0.302, 95%CI: 0.117–0.782). Greater gestational weight gain was associated with an increased risk of developing UI (OR: 1.030, 95%CI: 1.002–1.058). Moreover, higher economic income was associated with a higher likelihood of POP (OR: 2.335, 95%CI: 1.029–5.298). Furthermore, in our study, vaginal bacteria were found to be associated with abnormal fast-twitch muscle strength (OR: 2.311, 95%CI: 1.125–4.748). Finally, compared to primiparous women, multiparous women had an increased incidence of POP (OR: 3.892, 95%CI: 1.422–10.651). (Table 5).

**Table 5:** Binary regression analysis of risk factors related to pelvic floor muscle dysfunction.

	*Pre-rest*		*Post-rest*		*Fast muscle*		*Slow muscle stage*		*Urinary incontinence*		*Pelvic organ prolapse*	
	*stage*		*stage*		*stage*							
	OR	95%CI	OR	95%CI	OR	95%CI	OR	95%CI	OR	95%CI	OR	95%CI
**Model A**												
Maternal age ^a^	-	-	-	-	1.048	(1.001,1.098)	-	-	1.065	(1.015.1.116)	-	-
**Model B**												
Non-immigrant population ^b^	-	-	-	-	-	-	0.201	(0.046,0.872)	-	-	-	-
Maternal age ^b^	-	-	-	-	-	-	-	-	1.07	(1.019,1.123)	-	-
Prepregnant BMI ^b^	-	-	-	-	-	-	0.258	(0.088,0.759)	-	-	-	-
Constipation during pregnancy ^b^	1.622	(1.079,2.437)	1.47	(1.014,2.130)	0.644	(0.456,0.908)	0.435	(0.264,0.719)	-	-	-	-
**Model C**												
Non-immigrant population ^c^	-	-	-	-	-	-	0.211	(0.047,0.942)	-	-		-
Maternal age ^c^	-	-	0.937	(0.891,0.986)	1.073	(1.016,1.134)	1.109	(1.018, 1.209)	1.116	(1.052,1.183)	-	-
Constipation during pregnancy ^c^	1.553	(1.022,2.359)	-	-	0.652	(0.457, 0.930)	0.479	(0.285,0.804)	-	-	-	-
Mode of delivery ^c^	-	-	2.712	(1.189,6.185)	-	-	-	-	0.302	(0.117,0.782)		
Parity ^c^	-	-	-	-	-	-	-	-	-	-	3.892	(1.422, 10.651)
Weight gain during pregnancy ^c^	-	-	-	-	-	-	-	-	1.03	(1.002,1.058)	-	-
Economic income ^c^	-	-	-	-	-	-	-	-	-	-	2.335	(1.029,5.298)
Vaginal bacteria ^c^	-	-	-	-	2.424	(1.207, 4.867)	-	-			-	-

^a^ Adjusted for demographic data, including age, occupation, economic income, education level, whether immigrant population.

^b^ Adjusted for pregnancy related information based on model A, including pre-pregnancy body mass index, pregnancy diabetes, weight gain during pregnancy, pregnancy complications, constipation during pregnancy.

^c^ Adjusted for Postpartum related information based on model B, including delivery mode, perineal laceration, lateral episiotomy, vaginal pus cells, vaginal bacteria, gestational weeks, parity, fetal macrosomia. Only statistically significant data are shown in the table.

## Discussion

In this study, PFD was evaluated through both EMG and clinical diagnosis. Unlike previous studies, we identified additional factors associated with PFD beyond age, mode of delivery, gestational weight gain and parity. Specifically, constipation during pregnancy, immigrant status, economic income, and vaginal flora were found to be significant factors influencing PFD outcomes.

Several demographic variables were found to be associated with PFD. Age emerged as a significant factor influencing the risk of PFD, potentially associated with a decline in the passive mechanical performance of the PFMs [17]. Increased stiffness of the PFM had been reported to impair their function, affecting load-bearing capacity, contraction ability, and regenerative potential. Our study also indicated a higher likelihood of PFM dysfunction among immigrant populations. The term “floating population” referred to individuals who resided in an area without local registration, often for residential or occupational purposes. Many immigrants experienced increased physical strain and muscle fatigue due to efforts to augment their income and sustain their livelihoods [18]. Likewise, individuals of higher economic status may face heavier workloads and insufficient postpartum rest, which could increase their susceptibility to UI and other PFDs.

One study identified parity and constipation as potential risk factors for pelvic floor disorders [19]. In our research, we found that constipation during pregnancy was associated with abnormalities in postpartum resting PFM function. Constipation also demonstrated a protective effect on both fast-twitch and slow-twitch muscles. Constipation referred to the passage of stiff stools that could exert pressure on the internal pelvic muscles and organs during defecation [20]. Additionally, parity was recognized as a factor influencing pelvic floor function [21]. However, some studies reported that pelvic floor disorders were not significantly associated with the number of pregnancies and deliveries [22]. What’s more, several ultrasound-based studies found that pelvic floor discorders and structural deformation were unrelated to parity [23]. Therefore, further investigation is needed to clarify the impact of parity on pelvic floor function.

Previous research has indicated a correlation between gestational weight gain and ultrasonic parameters of the female pelvic floor during the early postpartum period [24]. This finding was consistent with the outcomes of our study, showing that gestational weight gain was correlated with fatigue in the PFMs. This correlation was attributed to heightened intra-abdominal pressure [25]. Furthermore, as weight increased, there was a risk of exceeding the endurance contraction amplitude threshold of the PFMs, potentially leading PFM dysfunction [26].

The mode of delivery, whether vaginal or cesarean, played a significant role in PFD. In our study, vaginal delivery was associated with a higher incidence of UI compared to cesarean delivery. During pregnancy, the enlarged uterus shifted its center of gravity forward, compressing the tissues of the pelvic floor [27]. During the vaginal delivery, the PFMs were extremely stretched, and muscle tissue, fascia, and ligaments were ruptured and damaged, potentially causing permanent nerve damage in some women [28]. Therefore, muscle fiber strength was more likely to be abnormal in women who delivered vaginally, increasing their risk of developing UI [29]. Another study reported a higher cumulative incidence of UI and overactive bladder in women who had vaginal deliveries compared to those who underwent cesarean sections [30]. However, cesarean delivery did not completely prevent the development of PFD. Compared to vaginal delivery, cesarean section led to abnormal resting muscle strength due to altered anatomical position between the bladder and uterus, which weakened the fascia supporting the bladder neck [31]. This resulted in PFM dysfunction during the resting stage. Moreover, the stress response following a cesarean section resulted in elevated inflammatory markers, ultimately contributing to PFD. Therefore, regardless of the mode of delivery, it was necessary to monitor postpartum pelvic floor function of the parturient.

This investigation has revealed a correlation between vaginal inflammation and PFD. We found upregulation of multiple immune-related gene ontology in multiple pelvic floor tissues of patients with POP and animal models, suggesting an inflammatory environment [32]. Specifically, mRNA expression levels of interferon gamma (IFNγ), IFN gamma receptor (IFNGR) and IFNGR2 were elevated in uterine tissue from patients with POP, along with upregulation of genes involved in the JAK-STAT signaling pathway, which activated IFNγ [33]. The inflammatory cytokine leakage process may disrupt the fibrous microstructure of pelvic floor tissues, potentially contributing to increased levels of vaginal flora in PFD.

This study employed a dual approach to evaluate pelvic floor muscle function, utilizing sensitive electrophysiology alongside clinical-specific diagnosis. The research were relatively comprehensive, yet it also had many limitations. It was conducted as a cross-sectional study, lacking further follow-up on maternal PFM function in postpartum women. In addition, abnormal electrophysiology was defined in this study as any deviation from normal criteria, leading to many women with abnormal EMG being asymptomatic. Further research is needed to determine whether conditions such as uterine prolapse or UI may develop later as a result.

## Conclusion

Among postpartum women, POP and UI were less common, but there were higher occurrences of abnormal electrophysiological values at each stage. In addition to uncontrollable factors like mode of delivery, age, and parity, interventions targeting weight gain and constipation during pregnancy and vaginal flora could mitigate the risks of PFD. Educational programs for pregnant women should provide guidance on a proper diet and lifestyle. Clinical treatment for women with vaginal inflammation should be initiated promptly to prevent exacerbating damage to the PFMs.

## Supporting information

S1 Table Basic information survey form. (PDF)
