# Supplementary material for: Pelvic floor dysfunction in postpartum women: A cross-sectional study
Source: PLoS One. 2024 Oct 3;19(10):e0308563. doi: 10.1371/journal.pone.0308563 (PMC11449369; doi:10.1371/journal.pone.0308563)
Supplement: S1 Table — (PDF) [file pone.0308563.s001.pdf]

| **1. Basic maternal information（Maternity registration number____________，____________ Hospital）** | | | |
|---|---|---|---|
| **Name** | **Telephone** | **A1 Age** | _________Years |
| **A2 Place of residence** | 1.Village；2. Cities and towns；3. County；4. Suburb；5. Urban area；6. Uncertain | | |
| **A3 Nation** | 1.Ethnic Han；2.Ethnic minorities（____ nationality） | | |
| **A4 Migrant population** | 1. Yes；2. No； | | |
| **A5 Occupation** | 1.Unemployment；2.Worker；3.Farmer；4.Individual operation；5.Enterprise and public institution；6.Athletes | | |
| **A6 Monthly income** | 1.Per capita ≤2000 yuan/month（ ）； 2.Per capita 2000-3000 yuan/month（ ）；<br>3.Per capita 3000-5000 yuan/month（ ）； 4.Per capita ≥5000 yuan/month（ ） | | |
| **A7 Degree of education** | 1.Junior high school and below；2.High School/Technical Secondary School；3.Undergraduate/College；4.Master's degree or above | | |
| **A8 Mode of delivery** | 1.Natural labor 2. Cesarean section | | |
| **A9 Marital status** | 1.Unmarried；2.Married 3.Divorced | | |
| **A10 Antecedent history** | 1.History of pelvic floor dysfunction related to: antenatal urinary leakage, antenatal urinary frequency, antenatal urinary incontinence, antenatal organ prolapse, antenatal constipation, antenatal low back pain<br>2.History of first vaginal delivery at advanced age<br>3.History of lower abdominal (pelvic floor) surgery: history of hysterectomy, laparoscopic and hysteroscopic surgery, uterine curettage, surgery for urinary incontinence, history of cesarean delivery<br>4.History of perineal lacerations<br>5.History of episiotomy<br>6.History of megabirths<br>7.Other（ ） | | |
| **A11 Current medical history** | 1.Postpartum urine leakage<br>2.Frequent postnatal urination<br>3.Postpartum organ prolapse<br>4.Postnatal constipation<br>5.Postpartum low back pain<br>6.Gestational diabetes<br>7.Diabetes （ ）Years<br>8.Recurrent urinary tract infections<br>9.Connective tissue disease | | |
| **A12 Family history** | 1.None 2.Yes（Diseases related to pelvic floor dysfunction, such as____________________） | | |
| **A13 Vaginal environment** | 1. Pyocyte 1.None；2.a little；3 +；4 ++；5 +++；6 ++++(not fill）<br>2. Bacteria 1.None；2.a little；3 +；4 ++；5 +++；6 ++++(not fill） | | |

| | | | |
|---|---|---|---|
| | Previous vaginitis: 1.None 2.Yes | | |
| **A14 Dietary habit** | 1.Strong tea None（ ） Yes （ ）cup/day<br>2.Coffee None（ ） Yes （ ）cup/day | | |
| **2.Pregnancy information** | | | |
| **B1 Pre-pregnancy weight** | ________kg | **B2 Current weight** | ________kg |
| **B3 Height** | ________cm | **B4 BMI** | Not fill |
| **B5 Weight gain in pregnancy** | ________kg | **B6 Using pelvic belt during pregnancy** | 1.None 2.Yes |
| **B7 Smoke** | 1.None 2.Sucked () | **B8 Constipation** | 1.None 2.Yes Bowel movements () once a week |
| **B9 Drink** | 1.Yes capacity for alcohol （ ） 2.None | | |
| **B10 Pregnancy complications or comorbidities** | 1.Yes（Designation ） 2.None（） | | |
| **3．Delivery status** | | | |
| **C1 Gestation** | ________Times | **C2 Parity** | ________Times |
| **C3 First gestational week** | ________Days | **C4 Gestational week of second birth** | ________Days |
| **C5 Age of first delivery** | ________Years | **C6 Mode of primary delivery** | 1.Natural labor 2.Vaginal delivery after induced labor 3.Cesarean section |
| **C7 Using pelvic belt during pregnancy** | 1.None<br>2.Yes（ ）（ ）<br>Hour/Day | **C8 The number of vaginal births** | ________Time |
| ***Vaginal delivery filling (medical record acquisition)*** | | | |
| **D1 Mode of delivery** | 1.Natural labor 2.Vaginal delivery after induced labor | **D2 Vaginal delivery** | 1.None（） 2.Yes（） |
| **D3 Total stage of labor** | ___________min | **D4 Second stage of labor** | ___________min |
| **D5 Oxytocin use** | 1.None（） 2.Yes（） | **D6 Laceration of perineum** | 1.None（） 2.Yes（） |
| **D7Lateral episiotomy** | 1.None（） 2.Yes（） | | |
| ***Cesarean section filling (Access to medical records)*** | | | |
| **E1Mode of delivery** | 1.Elective cesarean section and no labor 2.Cesarean section directly after delivery 3.Vaginal delivery by cesarean section | | |
| **E2Method of intraoperative anesthesia** | 1.General anesthesia；2.Continuous epidural anesthesia；3.Spinal anesthesia；3.General anesthesia + Continuous epidural anesthesia；4.Continuous epidural anesthesia + Spinal anesthesia | | |
| **E3Oxytocin was** | 1.None（） 2.Yes（） | | |

| | | | |
|---|---|---|---|
| **used after surgery** | | | |
| **5．Basic information of newborns** | | | |
| Newborns (access to medical records) | | | |
| **F1 Parity** | _________Times | **F2 Fetal position** | _________ |
| **F3 Neonatal birth weight** | _________g | | |
| **F4 Twins** | 1.None（） 2.Yes（） | **F5 Fetal macrosomia** | 1.None（） 2.Yes（） |
| **6．Pelvic floor muscle Screening (Access to medical records)** | | | |
| G1.Pre-resting stage | _________Points | G2.The post-resting stage | _________Points |
| G3.Fast muscles (Class 2) | _________Points | G4.Slow muscle (Class 1) | _________Points |
| G5.Total points | _________Points | G6.Abdominal muscle engagement | _________Points |
